# Impact of storage conditions on the shelf life of aflatoxin biocontrol products containing atoxigenic isolates of *Aspergillus flavus* as active ingredient applied in various countries in Africa

**DOI:** 10.1186/s43170-024-00283-6

**Published:** 2024-09-10

**Authors:** Alejandro Ortega-Beltran, M. O. Samuel Aikore, Lawrence Kaptoge, Daniel Agbetiameh, Juan Moral, Ranajit Bandyopadhyay

**Affiliations:** 1 International Institute of Tropical Agriculture, Ibadan, Nigeria; 2 Department of Crop and Soil Sciences, Kwame Nkrumah University of Science and Technology, Kumasi, Ghana; 3 Departamento de Agronomía, Universidad de Córdoba, Maria de Maeztu Unit of Excelente, Córdoba, Spain

**Keywords:** Bioprotectant, Long-term stability, Atoxigenic, *Aspergillus flavus*, Aflatoxin control

## Abstract

Aflatoxin contamination significantly threatens food safety and security, particularly in tropical and sub-tropical regions where staple crops such as maize, groundnut, and sorghum become frequently affected. This contamination is primarily caused by the fungus *Aspergillus flavus*. The contamination causes adverse health effects, reduced income, and trade restrictions. In response to this challenge, various technologies have been developed to mitigate the impacts of aflatoxin. Among these, biocontrol products containing atoxigenic isolates of *A. flavus* as the active ingredient can effectively reduce aflatoxin levels both at pre- and post-harvest. A notable example of such products is Aflasafe, which contains four atoxigenic isolates native to specific target regions. These products have undergone rigorous testing, have received regulatory approval, and are commercially available in multiple African countries. However, their manufacturing processes have evolved, and comprehensive shelf life studies for current formulations are lacking. Evaluations of the spore production ability of atoxigenic *A. flavus* isolates in Aflasafe products over 4 years, under various storage conditions, revealed a significant linear decrease in sporulation with storage months (*P* < 0.001; *R*^*2*^ = 0.203), with no significant differences observed between treatments. However, this marginal decline (*P* = 0.398) is unlikely to be sufficient to prevent the effectiveness in limiting aflatoxin. In addition, storing the products for 2 weeks at 54 °C did not affect (*P* > 0.05) the ability of the coated fungi to produce spores compared to when the products were stored at 24 °C. The findings contribute valuable insights for manufacturers and users of atoxigenic-based aflatoxin biocontrol products, informing best practices for product storage and utilization to ensure prolonged effectivenes in aflatoxin mitigation efforts.

## Introduction

In tropical and sub-tropical regions, maize, groundnut, and sorghum, among other crops, are frequently contaminated with aflatoxins by *Aspergillus* section Flavi fungi (Cotty et al. 1994; Udomkun et al. 2017). Aflatoxins are highly toxic carcinogenic compounds that negatively affect the health, income, trade, and development sectors (Dai et al. 2017; JECFA 2018; Logrieco et al. 2018). The Food and Agriculture Organization (FAO) estimates that up to 25% of the world’s susceptible crops contain mycotoxins above tolerance thresholds (FAO 2020). Additionally, it has been calculated that 60–80% of the susceptible crops contain multiple mycotoxins, even if not surpassing the tolerance thresholds (Eskola et al. 2020).

Unfortunately, human populations residing in aflatoxin-prone areas and relying on susceptible crops are chronically exposed to aflatoxins (Probst et al. 2007; Ezekiel et al. 2014; Kamala et al. 2018). Chronic exposure has been linked to liver cancer, stunted growth in children, and immunosuppression (Saha Turna and Wu 2023). The economic impact is highly severe, with aflatoxin contamination leading to important losses in crop value, trade restrictions, loss of income, and increased healthcare costs (Wu 2015; Ismail et al. 2021).

Globally, most aflatoxin contamination is attributed to *A. flavus* (Klich 2007; Amaike and Keller 2011). However, *A. flavus* genotypes that cannot produce aflatoxins (i.e., atoxigenic) are relatively common (Mehl et al. 2012; Sarrocco et al. 2019). The inability to produce aflatoxins is due to naturally occurring defects in genes necessary for aflatoxin production (Chang et al. 2005; Adhikari et al. 2016).

There are substantial research efforts to reduce incidence and severity of contamination events (Logrieco et al. 2018; Ojiambo et al. 2018; Matumba et al. 2021). The goal of those efforts is to prevent human and animal exposure and decrease agricultural losses and accompanying negative effects (Campbell et al. 2003; Hell et al. 2008; Waliyar et al. 2008; Ayalew et al. 2017; Seetha et al. 2017). A practical solution to limit aflatoxin contamination is to use atoxigenic isolates of *A. flavus* as biocontrol agents to displace aflatoxin producers in the field competitively (Dorner 2004; Cotty et al. 2007; Bandyopadhyay et al. 2016; Weaver and Abbas 2019). Atoxigenic isolates of *A. flavus* can be manufactured into biocontrol products for farmers to apply in the field. This type of product is typically applied 2–3 weeks before crop flowering. One application significantly reduces the proportion of aflatoxin producers in treated fields and considerably reduces the aflatoxins accumulated in treated crops compared to adjacent, untreated crops (Dorner 2004; Cotty et al. 2008; Doster et al. 2014; Weaver et al. 2015; Mauro et al. 2018; Bandyopadhyay et al. 2019; Senghor et al. 2020).

The atoxigenic-based aflatoxin biocontrol technology was developed by the United States Department of Agriculture – Agricultural Research Service (USDA-ARS) and registered with the United States Environmental Protection Agency (USEPA) for use in cottonseed in the US (Cotty et al. 2007). After conducting adaptive research, the technology was approved for use in maize, groundnut, pistachio, almond, and fig (Dorner 2009; Doster et al. 2014; USEPA 2016; Ortega-Beltran et al. 2019). This technology is now used outside the US, notably in various countries across sub-Saharan Africa (SSA). The International Institute of Tropical Agriculture (IITA) and USDA-ARS adapted and improved the technology for use in SSA in collaboration with local and international partners.

Several products under the tradename Aflasafe have been developed, each containing four atoxigenic isolates of *A. flavus* native to a target country as the active ingredients (Bandyopadhyay et al. 2016, 2022; Moral et al. 2020). High effectiveness of Aflasafe products in hundreds of thousands of hectares of farmlands cropped to maize, groundnut, sorghum, and chilies has been reported in Nigeria, Ghana, Senegal, Tanzania, and The Gambia (Bandyopadhyay et al. 2019; Ezekiel et al. 2019; Agbetiameh et al. 2020; Senghor et al. 2020, 2021) and in many countries for which scientific publications are not yet available (i.e., Burkina Faso, Burundi, DR Congo, Kenya, Malawi, Mali, Mozambique, Niger, Rwanda, Togo, Sudan, Uganda, Zambia). In general, the use of atoxigenic-based aflatoxin biocontrol products allows farmers to produce crops with 80–100% less aflatoxins than crops receiving no treatment. This results in the production of safer crops and the possibility of entering premium aflatoxin-conscious markets, which are very difficult to access because of low tolerance thresholds.

Aflasafe products are manufactured industrially by coating heat-killed, sterile sorghum grains with a suspension containing spores of the four atoxigenic isolates composing the product (Agbetiameh et al. 2019; Bandyopadhyay et al. 2019). The coating process is performed with a seed treater and facilitated by a polymer that helps the active ingredients adhere to the grains surface uniformly. In addition, a blue food colorant is added to differentiate the product from regular sorghum grain. After coating, the products are hermetically packaged in plastic bags containing 2, 2.5, 4, or 5 kg of product. Before the industrial process that started in 2013, a laboratory-scale method was used, in which batches of autoclaved sorghum grains were independently inoculated with a spore suspension of each atoxigenic isolate, incubated for 18 h at 31 °C, dried for 4 d at 50 °C in cloth bags, and thereafter combined and packaged (Atehnkeng et al. 2014). When the laboratory-scale method was used, the active ingredient fungi in the formulation remained viable for well over 2-years (Atehnkeng, J., *unpublished results*).

The viability of the active ingredient fungi in biocontrol products manufactured via the industrial process described above has not been reported. Therefore, the objectives of the current study were to assess the sporulation abilities of atoxigenic isolates of *A. flavus* composing aflatoxin biocontrol products (i) throughout 4 years of storage and (ii) after storage at a relatively high temperature. The results indicate that the spores of the evaluated atoxigenic isolates of *A. flavus* composing aflatoxin biocontrol products remain viable for 4 years regardless of the storage conditions and that a relatively high temperature had no negative influence on the sporulation ability of the biocontrol fungi. The results are valuable for current and future manufacturers of atoxigenic-based aflatoxin biocontrol products and users of the technology that purchase products and may need to store them for multiple years.

## Materials and methods

### Aflatoxin biocontrol products evaluated

Two products were evaluated: the aflatoxin biocontrol product developed for use in Nigeria, Aflasafe, and one of the two products developed for use in Ghana, Aflasafe GH01. Aflasafe contains four atoxigenic *A. flavus* genotypes (Og0222, La3279, La3304, and Ka16127) native to major agricultural areas in Nigeria (Atehnkeng et al. 2016). It has been registered with the Nigeria national regulator, Nigeria Agency for Food and Drug Administration and Control (NAFDAC), since 2014 (Bandyopadhyay et al. 2016). Aflasafe GH01 contains four atoxigenic *A. flavus* genotypes (GHG079-4, GHG083-4, GHG321-2, and GHM174-1) native to major agricultural areas in Ghana. It has been registered with Ghana Environmental Protection Agency since 2018 for use in maize, groundnut, and sorghum (Agbetiameh et al. 2020). The shelf life of the product developed for Nigeria was tested over a 4-year period. The product developed for Ghana was tested at a high temperature during a 2-week period.

### Manufacturing of biocontrol products

Mother cultures of each of the four atoxigenic isolates of *A. flavus* composing Aflasafe and Aflasafe GH01 are maintained in silica grains in IITA-Ibadan. Working cultures were obtained by plating a silica grain of each atoxigenic genotype on 5–2 agar [5% V8 Juice (Campbell Soup Company, Camden, NJ, US), 2% Bacto-agar (Difco Laboratories, Detroit, MI, US), pH 6.0)] and incubated for 5 d at 31 °C. Spores were dislodged using a cell spreader and suspended in 0.1% TWEEN®80. Each suspension was adjusted to 10^6^ spores/ml using a turbidimeter (Atehnkeng et al. 2014).

The production of billions of spores of atoxigenic fungi has been previously reported (Agbetiameh et al. 2019). Briefly, 4 ml spore suspensions from the working cultures of each atoxigenic isolate’s working cultures were independently inoculated in bottles containing 30 g of dead autoclaved sorghum grains and incubated for 7 d (31 °C, dark). The number of inoculated bottles per atoxigenic isolate depends on the quantity (tons) of biocontrol to be produced. For the biocontrol products manufactured and used in the current study, 24 bottles were inoculated with each atoxigenic isolate to produce 6 tons of each product. For each ton, a spore suspension (10 l, 4 × 10^7^ spores/ml) of the constituent atoxigenic isolates was combined with 1.5 l polymer (Sentry™, Precision Laboratories, Waukegan, IL, US) and 2 l blue non-toxic dye (Prism™, Milliken and Company, Spartanburg, SC, US), and coated on roasted, sterile sorghum grains (1 ton) with a seed treater (Bandyopadhyay et al. 2016). The biocontrol products were manufactured on 2 July 2016. Once manufactured, the products were placed in 2.5-kg corresponding plastic bags, hermetically sealed, and taken to the corresponding storage location (see below).

### Conditions of storage for the long-term study

Ten packages of 2.5 kg of the biocontrol product for Nigeria were randomly selected from the batches produced in the factory and stored in three different places: a cool storage room (~ 16 °C), a laboratory cabinet (~ 24 °C), and a storage area in the manufacturing facility (~ 30 °C). These storage conditions were chosen to simulate scenarios where distributors might store agricultural products before selling them to farmers. The product was stored in 2.5 kg plastic bags or 2.5 kg clear polythene bags in each place; the plastic bags contain a fine layer of polyethene film while the main component of the polythene bags is a thick layer of polyethene film. In all cases, the bags were placed inside cardboard boxes to prevent light exposure. Three replicated bags were evaluated for each type of plastic bag in each of the three places (cool room, laboratory cabinet, and general storage).

### Monthly sporulation evaluations

Spore production was evaluated monthly, on or around the 10th day of each month, from September 2016 to August 2020. Bags containing the product were brought inside a type 2 biosafety cabinet, aseptically opened, and grains were randomly collected and placed on sterile 96-well polystyrene plates (Thermo Fisher Scientific Inc., Waltham, MA, US) with the aid of sterile forceps. The plates were divided into four equal sections (24 wells each), each serving as one of the five repetitions for a treatment. Treatments were randomly placed across plates. For each repetition, a single grain of biocontrol product was placed in each of the 24 wells. Afterward, the bags were hermetically sealed and immediately returned to their respective storage conditions.

There were 18 plates used per monthly evaluation. Sterile water was added to the spaces between and outside the wells to maintain high humidity. The plates were randomly placed inside an incubator for 7 d at 31 °C. After incubation, the plates were visually inspected, and the number of kernels with visible *A. flavus* growth was recorded. Biocontrol grains with other microorganisms’ growth and other observations, such as kernel germination, were recorded.

For each treatment repetition, eight grains with *A. flavus* growth were randomly selected and used to quantify spore production. Ten ml 100% ethanol was used to wash the spores from each kernel pair, and combined with 10 ml distilled water in a sterile 25 ml glass cylinder. Spore production was quantified using a turbidimeter as previously described (Atehnkeng et al. 2014). Then, the average spore number values of the eight grains were calculated to compute the value for individual repetitions.

### Influence of temperature during a two-week period

The biocontrol product developed for Ghana was stored at 54 °C for 14 d. This test was required by Ghana Environmental Protection Agency as part of the tests needed to register biocontrol products of any kind for use in Ghana. Three batches of the biocontrol product manufactured as described above were used for this experiment. Two samples, each of 100 g, were collected from each batch. One sample of each batch was incubated in a laboratory oven at 54 °C for 14 d. The other sample was kept at room temperature (~ 24 °C) in a laboratory cabinet over the same period. Thereafter, the biocontrol grains were plated and incubated as described above. Each batch was replicated three times. Spore production was quantified as described above on the 3rd and 7th day after incubation. The experiment was repeated a second time using grains from batches used in the first experiment.

### Statistical analysis

To estimate the proportion of variability explained by the storage place (laboratory cabinet, cool room, and general storage area), the type of bag (plastic vs polythene), and the evaluation time, a General Linear Model (GLM) was performed on the sporulation data. We then assessed the contribution of the treatment, year, type of bag, and the different interactions according to Cohen’s f factor (Cohen 1988). Considering that: (i) the type of bags was not significant (*P* = 0.762); (ii) the effects of the different interactions were low or medium–low (Cohen’s f factor < 0.23); and (iii) we were more interested in comparing the storage locations than interactions; we performed a Friedman’s test. This latter is a nonparametric test that does not consider the interactions; i.e., storage room and evaluation times (41) were independently compared. The means were then compared using Dunn’s test with a Bonferroni adjustment (*P* ≤ 0.05) (Demšar 2006). Finally, for each storage place, linear regression was used to evaluate time over sporulation, and regression lines were compared based on the homogeneity of variances, slopes, and intercepts. The data were analyzed using Statistix 9 (Analytical Software) and SPSS v.19 (SPSS Inc., Chicago). For the experiment evaluating product samples stored at room temperature and 54 °C during a 2-week period, means were separated using paired Student *t*-tests (*P* ≤ 0.05) with SAS v9.2 (SAS Institute Inc., Cary, NC, US).

## Results

### Effect of long-term storage under different conditions on spore production

**Fig. 1:**
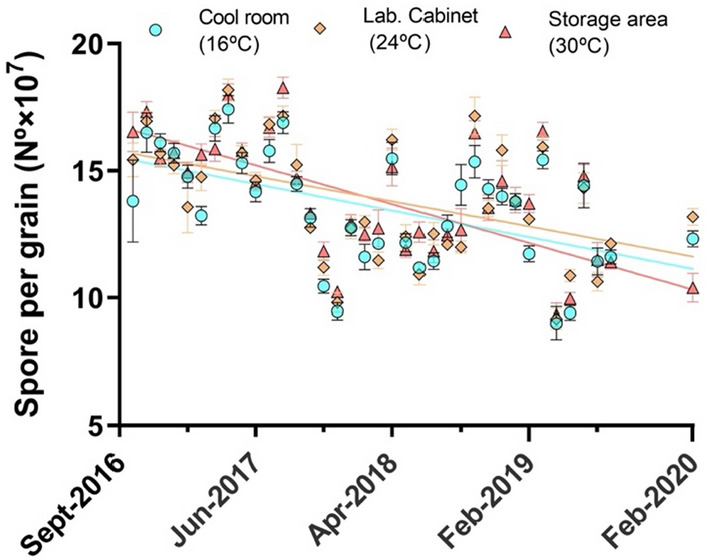
Variability in spore production per formulated grain by atoxigenic isolates of *Aspergillus flavus* in an aflatoxin biocontrol product evaluated during 4 years at three temperatures. Points represent the average of five data and vertical lines the SD. There was a significant linear decrease in sporulation with storage months (*P* < 0.001; *R*^*2*^ = 0.203)

The sporulation of a product stored at different temperatures and in two different plastic bags for up to 4 years was assessed. All biocontrol grains had sporulation of *A. flavus*, with none exhibiting fluffy growth. As expected, no other microorganisms were detected during the 4 years. However, the number of spores produced slowly decreased with storage time (*P* < 0.001), regardless of the temperature and bag type (Table 1; Fig. 1). In all cases, there was a significant linear decrease in sporulation with storage months (*P* < 0.001; *R*^*2*^ = 0.203), with no significant differences (*P* > 0.05) observed between treatments; i.e., the spore production ability of the coated fungi decreased gradually in all cases, but similarly.

**Table 1:** Analysis of variance for general linear models of data on spore production of an aflatoxin biocontrol product stored under different conditions and Cohen’s f factor

Source	Df	SS	Adj MC	F- value	*P*-value	Cohen's f Factor
Room	2	3.25E + 15	1.63E + 15	8.73	< 0.001	0.06
Type of bag	1	1.72E + 13	1.72E + 13	0.09	0.762	0.00
Evaluation data	40	6.96E + 17	1.74E + 16	93.33	< 0.001	1.57
Room*Bag	2	1.71E + 15	8.54E + 14	4.58	0.012	0.04
Room*Evaluation	80	5.24E + 16	6.55E + 14	3.51	< 0.001	0.23
Bag*Evaluation	40	1.68E + 16	4.21E + 14	2.26	< 0.001	0.13
Room*Bag*Evaluation	80	2.47E + 16	3.09E + 14	1.66	< 0.001	
Error	984	1.83E + 17	1.86E + 14			
Total	1229	9.78E + 17

**Fig. 2:**
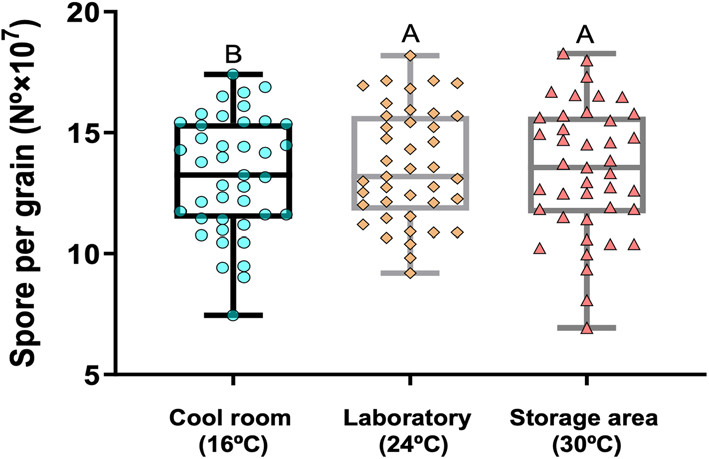
Spore production by the atoxigenic isolates of *Aspergillus flavus*  in formulated biocontrol grains stored at different temperatures during the 48 months of evaluation. The means were compared using Dunn’s test with a Bonferroni adjustment (*P* ≤ 0.05). Means with the same uppercase letter are not significantly different

The general linear model showed that the factor exerting the most influence (Cohen’s f factor = 1.57, considered highly significant) on grain sporulation was the evaluation time (Table 1). The rest of the interactions were significant (*P* < 0.05), but with low or very-low impact on the sporulation according to the Cohen’s f factor (f ≤ 0.23). When examining the storage temperature independently according to the Friedman’s test, there were differences, although minimal, in spore production in products stored at different temperatures (Fig. 2). Overall, greater (*P* < 0.05) spore production occurred in products stored at 24 °C and 30 °C than in those stored at 16 °C (Fig. 2). Further, products stored in plastic bags produced slightly more spores than those stored in polythene bags, although those differences were not significant (*P* = 0.762; Table 1).

### Effect of a relatively high temperature during two weeks on spore production

**Fig. 3:**
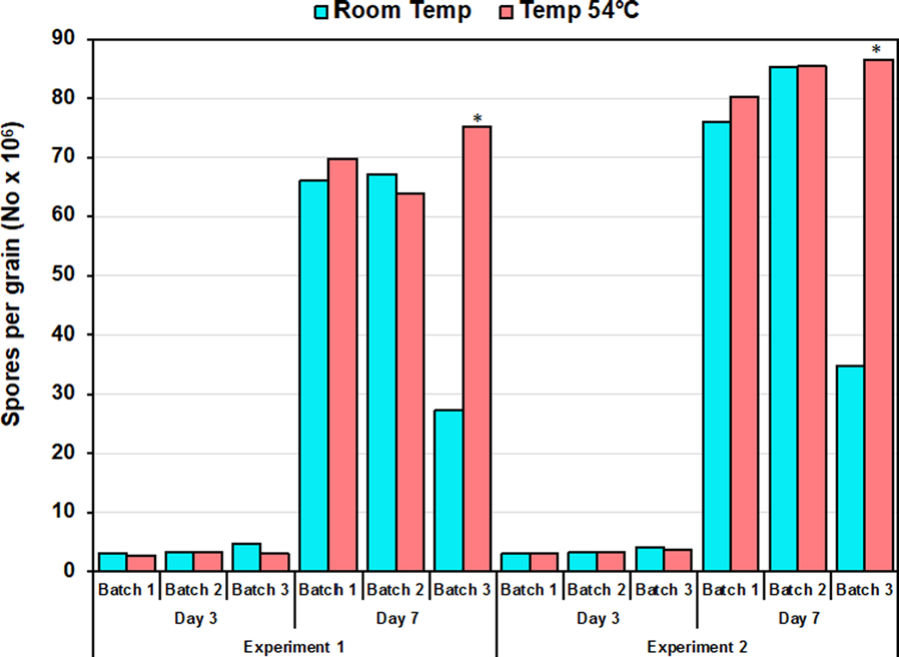
Spore production by the atoxigenic isolates of *Aspergillus flavus*  in formulated biocontrol grains stored at room temperature (~ 24 °C) and 54 °C for 14 days. After the storage time, spore production was evaluated at the 3rd and 7th day of incubation at 31 °C. In each comparison, the means were separated using paired Student *t*-tests (α = 0.5). Bars with an asterisk (*) indicate differences in sporulation. Differences were found only in products from Batch 3, at Day 7, in both experiments

Relatively low sporulation occurred in batches of the product incubated for three days, regardless of treatment, compared to those batches incubated for seven days (Fig. 3). Overall, 17- to 23-fold more sporulation occurred on day seven. On the third day of incubation, sporulation in products stored at room temperature (24 °C) was not significantly (*P* > 0.05) different from that in products stored at 54 °C (Fig. 3). However, on day seventh in both experiments, in one of the batches greater (*P* < 0.05) sporulation occurred after storage at the highest temperature, 54 °C (Fig. 3). Grains from the batch incubated at room temperature had a whitish fluffy mass growth which inhibited sporulation on the grains. The sporulation in the other two batches was statistically similar, and no fluffy growth was observed in any of the evaluated grains in these treatments.

## Discussion

In sub-Saharan Africa, comprehensive, integrated management strategies are needed for current and severe aflatoxin contamination events in staple crops (e.g., maize, groundnut, sorghum) (Ayalew et al. 2017; Matumba et al. 2021). A technology proven effective in controlling aflatoxins from field to plate is biocontrol, which involves the use of atoxigenic isolates of *A. flavus* as active ingredients (Bandyopadhyay et al. 2022). Although several atoxigenic-based products are commercially available in several African countries, there is limited information on their shelf life and appropriate conditions for storage. The current study provides evidence of the long-term shelf life of aflatoxin biocontrol products manufactured with industrial processes currently used in various countries. The results indicate that a high spore production capacity remains after 4 years of storage and that refrigerated storage is not required to maintain a high spore production capacity. We also found that the spore production ability of atoxigenic isolates of *A. flavus* was not affected when the products containing them were stored at a relatively high temperature, 54 °C, for a 2-week period. The results provide valuable information to manufacturers, distributors, and users of the technology. Products stored for multiple years remain viable and can protect crops from aflatoxin contamination well over the initial recommendation of 2 years of storage.

In general, the use of fungi as active ingredients in bioprotectants faces several challenges, including difficulties in mass producing both the active ingredient and the product itself, a reduced shelf life, and inconsistent field performance (Bateman 2004; Jaronski and Mascarin 2013; de la Cruz Quiroz et al. 2019). Fortunately, the atoxigenic-based biocontrol technology is not affected by such challenges. The production of the active ingredient fungi is relatively easy and there is no need for large equipment (Ortega-Beltran et al. 2022; Senghor et al. 2021); the products are effective at limiting aflatoxin (on an average 80–100% less aflatoxin compared to untreated crops) across largely contrasting cropping systems in both subsistence and commercial agriculture (Bandyopadhyay et al. 2019; Agbetiameh et al. 2020; Senghor et al. 2020; Mahuku et al. 2023; Ola et al. 2022; Ortega-Beltran et al. 2022), and at least 2 years of shelf life have been recorded for products manufactured using a wet, medium-scale process (Atehnkeng et al. 2014; Senghor et al. 2021). When registered (2014–2018), the products were manufactured using a process that differs from the one currently used, an evolved industrial process. The current study was conducted because it would be valuable to current and future manufacturers, distributors, and users of the technology to know for how long the products can be stored and under which conditions; finding biocontrol products with extended shelf life may increase chances of both manufacturing companies interested in investing in their production and increased adoption of the products (de la Cruz Quiroz et al. 2019). Ultimately, increased production and commercialization of biocontrol products to combat pests and diseases can significantly contribute to producinge crops with reduced carbon footprints in an environmentally sustainable manner.

The evaluation demonstrated the resilience of atoxigenic-based aflatoxin biocontrol products over extended periods. Of course, spore production is greater during the first few months after manufacturing. However, even after a 4-year period of storage, large numbers of spores are produced, and these would be sufficient to provide the required densities of atoxigenic fungi in the field to outcompete toxigenic fungi. Products are manufactured based on orders from customers, and storing them for more than 2 years is unlikely. However, if products have to be stored for more than 2 years, a natural decline (*P* < 0.001; R^2^ = 0.203) in spore production ability is not expected to reduce the product’s effectiveness. Refrigerated conditions are not required for prolonged storage of the Aflasafe products. Indeed, greater spore production was detected when the products were stored at 24 °C and 30 °C (Fig. 2). The long shelf life of atoxigenic-based aflatoxin biocontrol products is significant considering the potential need for extended storage periods depending on the ability of distributors to sell the products. However, while the products demonstrated sustained potency over the study period, it is advisable to reduce the storage time as much as possible to prevent potential losses in effectiveness or product quality due to other circumstances, e.g., rodent or insect attack with subsequent microbial contamination and/or exposure to humidity. The observed decline in spore production ability may be attributed to reduced viability of spores as they age coupled with deterioration of the nutrients in the sorghum grain over time. These factors deserve further investigation.

In regions where distribution networks may be limited or market demand fluctuates, the ability to store these products for multiple years without compromising efficacy ensures continuous availability and accessibility. The prolonged shelf life mitigates concerns regarding product expiration and waste, allowing distributors to maintain sufficient stock levels to meet market demands. The  ability of storing the product without the need for large refrigeration chambers is an important cost-saving factor for the industries producing this biocontrol technology. Moreover, it enhances the feasibility of bulk purchasing and long-term storage strategies, offering cost-saving opportunities and logistical advantages for distributors and end-users. Overall, the extended shelf life of atoxigenic-based biocontrol products enhances their sustainability and utility in safeguarding food safety and security in aflatoxin-prone regions.

The spore production ability of the product after storage at 54 °C for 14 days was evaluated at the request of the regulator in Ghana. The results of this evaluation (Fig. 3) further confirmed the product stability even under such high temperature conditions. There may be occasions in which aflatoxin biocontrol products are transported in containers where temperatures are greater than 50 °C in regions where temperatures are high during several hours of the day. With the demonstrated stability of spore production after exposure to relatively high temperatures, concerns that may arise regarding product viability during transportation should be alleviated. Manufacturers, distributors, and users can confidently transport these products without the need for stringent temperature control measures, thereby streamlining distribution logistics and potentially reducing associated costs.

The current study also investigated the influence of different types of packaging. Interestingly, the type of bag used for storage had no significant effect on spore production capacity (Table 1). Whether stored in plastic bags or polythene bags, the biocontrol products maintained consistent spore production levels over the 4-year evaluation period. The choice of packaging material does not impact the viability or performance of the biocontrol products, providing manufacturers with flexible options for selecting bags.

It was not possible to conduct field effectiveness trials with products obtained from the evaluated treatments because the demand to evaluate products monthly for 4 years exceeds the limitations of cropping cycles everywhere. Aflatoxin biocontrol products are applied once per cropping season, 2–3 weeks before flowering (Bandyopadhyay et al. 2016). Thus, only one or two of the treatments could have been evaluated in a year. In the current study, spore production on the products in the monthly laboratory assays was utilized as a proxy for the potential effectiveness of the stored biocontrol products if applied in farmer fields. Products stored for long but showing high sporulation would eventually be effective in displacing aflatoxin producers in farmers’ fields. The spores coated onto the sorghum grains, although produced for the formulation process up to 4 years prior, would produce millions of newly formed spores during at least 20 days (Bandyopadhyay et al. 2022). The “old” spores would serve to start the production of biocontrol propagules in the field. The “new” spores would have the same potency as if produced from coated spores produced one day before.

## Conclusion

The comprehensive evaluation of atoxigenic-based aflatoxin biocontrol products over 4 years has provided valuable insights into the long-term shelf life and stability of these products under various storage conditions. The study revealed that these biocontrol products have remarkable resilience, maintaining consistent spore production levels irrespective of the storage temperature and packaging material. Further, the results suggest that stringent temperature control measures may not be necessary, particularly in regions with high ambient temperatures. These findings offer valuable guidance to manufacturers, distributors, and users of atoxigenic-based aflatoxin biocontrol products, both in areas where the products are used and in those where they will soon be used, facilitating informed decision-making and optimizing their use in aflatoxin management strategies.

## Data Availability

The datasets used and/or analysed during the current study are available from the corresponding author on reasonable request.
